# Snake Venom Disintegrins and Cell Migration

**DOI:** 10.3390/toxins2112606

**Published:** 2010-10-29

**Authors:** Heloisa S. Selistre-de-Araujo, Carmen L. S. Pontes, Cyntia F. Montenegro, Ana Carolina B. M. Martin

**Affiliations:** Departamento de Ciências Fisiológicas, Universidade Federal de São Carlos, São Carlos, SP, 13565-905, Brazil; Email: carmenpontes@yahoo.com.br (C.L.S.P.); cyntia_montenegro@hotmail.com (C.F.M.); carol_gau@yahoo.com.br (A.C.B.M.M.)

**Keywords:** cell migration, disintegrin, snake venom, ADAM, α_v_β_3_ integrin

## Abstract

Cell migration is a key process for the defense of pluricellular organisms against pathogens, and it involves a set of surface receptors acting in an ordered fashion to contribute directionality to the movement. Among these receptors are the integrins, which connect the cell cytoskeleton to the extracellular matrix components, thus playing a central role in cell migration. Integrin clustering at focal adhesions drives actin polymerization along the cell leading edge, resulting in polarity of cell movement. Therefore, small integrin-binding proteins such as the snake venom disintegrins that inhibit integrin-mediated cell adhesion are expected to inhibit cell migration. Here we review the current knowledge on disintegrin and disintegrin-like protein effects on cell migration and their potential use as pharmacological tools in anti-inflammatory therapy as well as in inhibition of metastatic invasion.

## 1. Introduction

Metastasis is one the major causes of death in patients with cancer. In order for a tumor to grow and metastasize, both tumor and endothelial cells must migrate and invade surrounding tissues [1,2]. Endothelial cell migration provides the blood supply that is essential to tumor cells. Once in the blood vessels, tumor cells must adhere to the endothelium and escape to a new site [3], two processes that depend on their invasive abilities. Therefore, the blockage of both tumor and endothelial cell migration and invasion is an interesting approach for the treatment of cancer patients. The key receptors involved in cell migration are the integrins, which connect the cells to the extracellular matrix of the tumor microenvironment. Integrin blocking usually results in inhibition of cell migration and tumor angiogenesis [4]. Here we review the effects of disintegrins, a group of integrin-binding proteins found in snake venoms, in the cell adhesion process and their application in anti-cancer and anti-metastatic therapy. The effects of disintegrin on neutrophil migration will also be briefly described for a better illustration of the role of these proteins in cell migration.

## 2. Integrins and Cell Migration

Integrins are cell surface receptors that play critical roles in cell adhesion and migration. These proteins are heterodimers of transmembrane α- and β-subunits that connect the extracellular matrix (ECM) to the cell cytoskeleton. Cell binding by integrins to their cognate extracellular ligands, such as collagen, laminin and fibronectin, triggers intracellular signaling pathways that control cytoskeleton organization, cell polarity and force generation [5]. Integrin clustering at focal complexes drives actin polymerization along the leading edge of migrating cells, contributing to changes in cell shape and polarity [6]. Integrins are also involved in the regulation of matrix-degrading proteases, a key step for the invasive phenotype. There are several excellent reviews on the role of integrins in cell migration and invasion [6,7,8], and therefore, this subject will not be addressed in the present review. Furthermore, several studies have described the correlation between a malignant phenotype and an altered integrin distribution on tumor cell surfaces [9]. The role of integrins in metastatic dissemination is also very well documented [10,11], and is beyond the scope of this review. 

## 3. Angiogenesis

The term angiogenesis usually defines the growth of sprouts from capillary blood vessels that depends on a delicate balance between pro- and anti-angiogenic factors. In mammals, physiological angiogenesis is restricted to specific situations, including growth, tissue regeneration and reproduction. In contrast, some angiogenesis-dependent diseases, such as tumor growth, age-related macular degeneration and atherosclerosis, have been described [12]. 

Angiogenesis is primarily driven by tissue hypoxia that upregulates hypoxic factor-1 (HF-1) expression [7,13], which in turn induces vascular endothelial cell growth factor (VEGF) expression, a potent mitogen for endothelial cells [7,14]. Briefly, the angiogenic cascade includes degradation of basement membrane (BM) by matrix metalloproteases (MMPs) expressed by endothelial cells (EC), EC proliferation and tube formation, synthesis of a new BM, tube stabilization by pericytes and vascular smooth muscle cells and maturation into capillaries [15]. In addition, extracellular matrix degradation by MMPs plays a key role in the control of angiogenesis either by releasing VEGF from the ECM and facilitating EC migration, or by releasing angiogenesis inhibitors from larger extracellular matrix components such as endostatin from collagen XVIII [16,17], and angiostatin from plasminogen [15,18]. Angiogenesis is also influenced by integrins expressed on endothelial cells, vascular smooth muscle cells, fibroblasts, and platelets. These cells process signals from their microenvironment and respond by altering their cell-cell and cell-matrix adhesion, which allows migration and vascular remodeling over a period of days to weeks [19]. 

On the other hand, tumor angiogenesis is not particularly effective. Tumor vessels are structurally and biologically different with leaky characteristics that facilitate tumor cell dissemination by blood or lymphatic vessels [4,20]. Unlike in quiescent EC, the α_v_β_3_ integrin is highly expressed by tumor EC, which helps the binding of new ECs to the provisional matrix components, including vitronectin and fibronectin that are deposited in the tumor microenvironment [4]. Accordingly, small integrin binding proteins have been tested for their ability to inhibit tumor angiogenesis. We will review the work that has been done with snake venom disintegrins in this field.

## 4. Disintegrins from Snake Venoms

Snake venom disintegrins are mostly derived from proteolytically processed precursor forms having a metalloprotease domain, named the snake venom metalloproteases (SVMPs). Members of this protein family have been classified according to their multi-domain structure into P-I, P-II and P-III classes [21]. Members of the P-I class are formed by a metalloprotease domain only while P-II proteins have a metalloproteinase and a disintegrin domain. P-III proteins have an additional cysteine-rich domain following the disintegrin region and in some cases, a lectin domain [22]. These last two classes (P-II and P-III) can be subdivided further according to the proteolytic processing of their domains and their ability to form dimeric structures [23]. Based on this classification, processed RGD-disintegrins are derived from the P-IIa class, while homo- and heterodimeric disintegrins are released in general from SVMPs belonging to the P-IId and P-IIe classes, respectively. P-III SVMPs originate the disintegrin-like proteins (DC, for Disintegrin, Cys-rich proteins) formed by covalently linked-disintegrin-like and Cys-rich domains [21]. By means of phylogenetic analysis, Calvete *et al.* [24] provided strong evidence that the diversity of disintegrins could be due to an accelerated evolution of surface-exposed residues with the inference that the RGD motif is the ancestral integrin-recognition motif from which other disintegrins have emerged via single-base substitutions to generate KGD-, MGD-, WGD-, and VGD-integrin binding motifs as well as others. The diversity of the integrin-binding loop, along with other conserved residues among disintegrin molecules, gives rise to different integrin specificities (Table 1). However, disintegrin structural complexity does not derive only from venom genomic structure and transcriptional regulation events but also from post-translational modifications that could be responsible for dimerization and disulfide bonding as previously suggested [21].

DC proteins are usually found in a processed form in snake venoms without the catalytic domain but they are not further separated into independent proteins due to a disulfide bond that connects both domains. The active RGD loop of P-II-derived disintegrins is modified into a cysteine-contained loop (D/ECD) in the DC proteins [25]. It has been recently suggested that distinct disulfide pairing strongly contributes to the conformation of the D domain, which could consequently influence the binding properties and specificities of DC proteins [26]. Depending on the disulfide pairing, the D domain may assume at least two types of conformation, a C-shaped or an I-shaped scaffold; the former was suggested to play a key role in substrate recognition by the catalytic domain [27]. The D domain also has adhesive properties to some proteins such as collagen I, as recently demonstrated. 

Integrin binding ability is apparently more related to the Cys-rich domain [28,29] which was also shown to bind von Willebrand factor therefore helping substrate targeting for proteolysis by the metalloprotease domain [30]. In addition, the hyper-variable region (HVR), considered the major structurally distinct region among the P-III SVMPs and suggested to play a key role in target selection due to its protein-protein adhesive properties, is located in the Cys-rich domain [29]. The importance of HVR was recently evidenced for two elapidic SVMPs from *Naja atra* venom. Atragin, a SVMP with a C-shaped D domain, but not its homolog K-like, which has an I-shaped scaffold, inhibits the migration of both mouse fibroblasts and Chinese hamster ovary CHOK1 cells [26]. Synthetic peptides from the HVR of both atragin and K-like proteins showed similar results, since only the peptide from atragin HVR inhibited cell migration toward fibronectin [26].

Disintegrins and DC proteins are rich in Cys residues, which are mainly involved in disulfide bonds, resulting in proteolysis-resistant molecules. This is a crucial feature for a sustained half-life in the blood. DisBa-01, a recombinant disintegrin from *Bothrops alternatus* venom, could be detected up to 6 h hours after i.p. injection in mice [31]. Despite the high content of disulfide bonds, RGD-disintegrins can be produced in an active form in bacteria [32,33], thus allowing the production of large quantities as needed for *in vivo* tests. Recombinant DC proteins are more difficult to express in an active form in bacteria. However, the production of Cys-rich domains from P-III SVMPs in active form has been reported [29]. 

## 5. Effects of Disintegrins on Leukocyte Migration

Neutrophils can be recruited from blood circulation toward sites of inflammation due to signals from injured tissues. To reach these sites, neutrophils must express new receptors, including some integrins, on their cell surface that will allow their adhesion to the endothelium and subsequent migration into tissues [34]. In addition to several constitutively expressed integrins such as α_M_β_2_, α_v_β_3_ and α_9_β_1_, these cells express α_2_-, α_3_-, α_4_-, and α_5_β_1_ integrins upon activation [35,36], triggering integrin-signaling pathways that mediate actin polymerization, cytoskeletal organization, spreading and migration [37]. Accordingly, disintegrins were first tested as integrin inhibitors of neutrophil migration as potential anti-inflammatory candidates. However, the results showed that integrin-binding by disintegrins could also activate neutrophils and protect these cells from apoptosis [38,39], demonstrating the complexity of disintegrin effects. 

Coelho *et al.* [38] first demonstrated the dual effect of the monomeric RGD-disintegrin jarastatin (JT), from *Bothrops jararaca* venom, on neutrophil chemotaxis. Jarastatin inhibited neutrophil migration toward fMLP, IL-8 and jarastatin itself in a concentration-dependent manner. However, JT also induced neutrophil chemotaxis when used as chemoattractant [38]. In addition, it was demonstrated that this disintegrin induced several downstream integrin-mediated signaling events such as actin polymerization, activation of focal adhesion kinase (FAK) and extracellular-regulated kinase-2 (erk-2) nuclear translocation, which resulted in a delay of spontaneous neutrophil apoptosis [40]. JT also inhibited neutrophil migration *in vivo* after intraperitoneal carrageenan injection [38]. Ocellatusin, an RGD-disintegrin from *Echis ocellatus* venom, also strongly induces neutrophil chemotaxis [41]. Later, the same group demonstrated that EC3, a heterodimeric MLD-disintegrin from *Echis carinatus* venom, also inhibited neutrophil chemotaxis and activated FAK and phosphoinositide 3-kinase (PI3K); however, in contrast to JT, EC3 inhibited Erk-2 translocation and had a pro-apoptotic effect [39]. Antibody competition assays showed that JT and EC3 effects are mediated by different neutrophil integrins. JT binds preferentially to α_M_β_2_ while EC3 is a ligand for α_9_β_1_ in neutrophils [39,42]. In contrast, it has been recently shown that activation of α_9_β_1_ integrin by VLO5, a dimeric disintegrin from *Vipera lebetina obtusa* venom [43], inhibits neutrophil spontaneous apoptosis by up-regulating the expression of anti-apoptotic proteins Bcl-x_L_ and by increasing the degradation of pro-apoptotic protein Bad [36]. The two dimeric disintegrins EC3 and VLO5 are an interesting example of how subtle the structural differences that lead to distinct biological effects can be. Both disintegrins are very similar and have the VGD motif on the A-subunit while the B-subunit has a single amino acid replacement N-terminally to the MLD adhesive motif. This MLD motif has been suggested to be responsible for the different binding specificities and biological effects. An alanine residue in EC3 (AMLD) is replaced in VOL5 by a threonine residue (TMLD), which could result in distinct affinities and effects, despite their binding to the same integrin receptor [36].

RGD-disintegrins were also demonstrated to activate human T lymphocytes via integrin signaling [44]. Flavoridin, from *Trimeresurus flavoviridis*, kistrin, from *Agkistrodon rhodostoma*, and echistatin, from *Echis carinatus* venoms, which all bind to α_5_β_1_ and α_v_β_3_, induced T cell proliferation and CD69 expression in parallel with FAK and PI3K activation and NF-κB nuclear translocation [44]. 

Alternagin-C (ALT-C), an ECD-containing disintegrin-like protein from *Bothrops alternatus* venom induced neutrophil effects similar to those of JT [45]. It inhibited neutrophil migration in a concentration-dependent fashion despite being itself chemotactic to this cell type. ALT-C also induced significant cytoskeleton dynamic changes with polymerization of F-actin, FAK and PI3K activation, and erk-2 translocation as well. These effects were reproduced by an ECD-containing peptide derived from the ALT-C primary sequence [45,46]. Interestingly, ALT-C was demonstrated to be an inhibitor of α_2_β_1_ integrin-mediated cell adhesion to collagen I. Jararhagin-C, the released DC domain from the SVMP jararhagin isolated from *Bothrops jararaca* venom, also induced *in vivo* leukocyte rolling after topical application on murine cremaster muscle. These observations together confirm the key role for the α_2_β_1_ integrin in neutrophil migration [47].

The complexity of the DC proteins was further demonstrated by the work of Menezes and co-authors, showing that the recombinant Cys-rich domain of HF3 metalloprotease from *Bothrops jararaca* venom is able to activate leukocyte rolling in the microcirculation [29]. This protein was expressed in bacteria without the disintegrin-like domain thus providing convincing evidence of that DC proteins activity resides on the Cys-rich region. In addition, synthetic peptides derived from the HVR region of the Cys-rich domain of HF3 reproduced the same effect, which was also inhibited by anti-α_M_β_2_ antibodies, confirming the integrin-mediated activity of the C domain in leukocytes. 

## 6. Effects of Disintegrins on Endothelial Cell Migration and Angiogenesis

Triflavin from *Trimeresurus flavoviridis* venom was one of the first RGD-disintegrins shown to inhibit angiogenesis both *in vitro* and *in vivo* [48]. Triflavin (0.4 μM) strongly inhibited EC migration toward vitronectin and fibronectin nearly thirty orders of magnitude greater than the anti-α_v_β_3_ monoclonal antibodies [48]. Triflavin was also more effective in inhibiting TNF-α-induced angiogenesis in the chicken chorioallantoic membrane (CAM) assay. Similar results were obtained with another RGD-disintegrin, rhodostomin, from *Agkistrodon rhodostoma* venom, which inhibits endothelial cell migration, invasion and tube formation evoked by bFGF in matrigel both *in vitro* and *in vitro* [49]. Rhodostomin effects were inhibited by anti-α_v_β_3_ but not by anti-α_v_β_5_ antibodies, thus supporting the hypothesis that the effects of RGD-disintegrins are mediated by blockade of the vitronectin receptor.

Native or recombinant contortrostatin (CN), a homodimeric RGD-disintegrin from *Agkistrodon contortrix contortrix* venom, is another example of anti-angiogenic disintegrin as demonstrated by several *in vitro* and *in vivo* models [50,51,52]. Liposomal delivery of CN has proven to be effective as an anti-angiogenic and anti-tumor agent in human ovarian and breast cancer animal models [52]. Similar results were obtained with vicrostatin, a quimeric recombinant CN variant, which was also demonstrated to induce apoptosis in tubulogenic HUVEC seeded between two matrigel layers [53]. A monomeric recombinant RGD-disintegrin from *Bothrops alternatus* venom, Dis*Ba*-01, produces similar effects in the matrigel plug model in nude mice [33]. In addition to inhibition of EC migration, saxatilin, an RGD disintegrin from *Gloydius saxatilis* also inhibited the migration of vitronectin-induced smooth muscle cells [54,55].

However, the complexity of disintegrin specificity and the process of migration do not allow for the conclusion that blocking the vitronectin receptor is sufficient for inhibiting cell migration. Echistatin, but not eristostatin (two short RGD-disintegrins), inhibits *in vivo* angiogenesis in the CAM assay [56]. There are at least three KTS-disintegrins that inhibit α_1_β_1_ integrin binding to its cognate ligands, collagen I and IV (Table 1) [57,58], and also inhibit EC migration toward these substrates [59]. Lebestatin is an example of a KTS-disintegrin isolated from *Macrovipera lebetina* that inhibits EC migration and VEGF-induced *in vivo* angiogenesis [59]. The presence of a WGD motif in CC8, a heterodimeric disintegrin from *Echis carinatus*, increases its inhibitory effect on α_v_β_3_ and α_5_β_1_ integrins [60]. 

There are few reports regarding the effects of ECD-disintegrins on endothelial cell migration. Acurhagin-C, an ECD-disintegrin-like protein from *Agkistrodon acutus* venom, dose-dependently blocked HUVEC migration toward a vitronectin-coated membrane. Furthermore, acurhagin-C elicited endothelial anoikis via disruption of the α_v_β_3_/FAK/PI3K survival cascade and subsequent initiation of the procaspase-3 apoptotic signaling pathway [61]. Similarly, ALT-C also inhibits EC migration both *in vitro* and *in vivo* [62,63]. 

## 7. Effects of Disintegrins on Tumor Cell Migration

The ability of most snake venom disintegrins to inhibit cell adhesion has stimulated scientists to study these proteins as inhibitors of tumor cell dissemination. A volume of papers dealing with this subject can be found in the literature, most on RGD-disintegrin effects (Table 1). Morris *et al.* [64], were one of the first investigators to test eristostatin, an RGD-disintegrin from *Eristocophis macmahoni*, on individual metastasis steps such as cell arrest, extravasation and migration. Eristostatin treatment did not prevent tumor cell extravasation or migration [55]. However, it was shown later that eristostatin inhibited melanoma cell motility, an effect mediated by fibronectin-binding integrins [56]. Interestingly, this disintegrin, contrary to other RGD-disintegrins, did not inhibit angiogenesis, as stated before [56]. 

**Figure 1:**
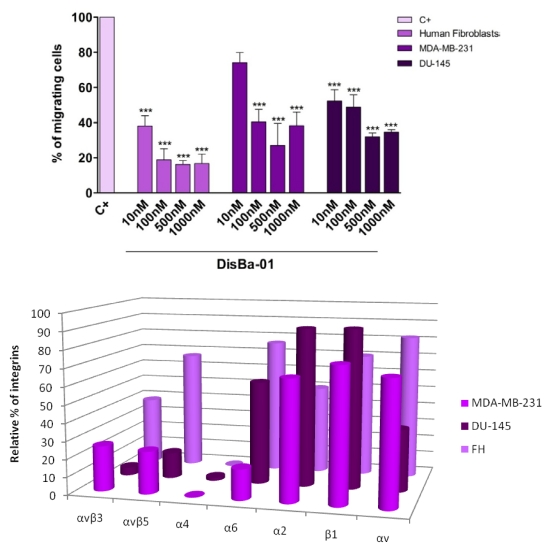
A (top) Effects of Disba-01 on the migration of three different cell lines. The cells were plated on the migration inserts in the presence of increasing concentrations of DisBa-01 for 22 h. Migration was expressed as a percentage of the control (100%). Cells were counted with a microscope (ten random fields per experiment). Results represents mean ± standard error of three individual experiments, *** p < 0.0001. **B** (bottom) Integrin profile of the cells tested in the migration assay using DisBa-01. Fibroblasts (FH) express α_v_β_3_, α_v_β_5_, α_6_, and β_1_. MDA-MB-231 breast tumor cells express α_v_β_3_, α_v_β_5_, α_6_, α_2_, and β_1_. DU-145 prostate tumor cells express more α_v_β_5_, α_6_, α_2_, β_1_, and less α_v_β_3_ and α_4_. The presence of integrin receptors on the cell surface was determined by flow cytometry with FITC-anti-α_v_β_3_, α_v_β_5_, α_6_, α_4_, α_2_, β_1_ and α_v_ subunit integrin antibodies.

DisBa-01, an α_v_β_3_ integrin-blocking RGD-disintegrin, inhibits not only endothelial cell *in vivo* migration [33] but also the *in vitro* migratory ability of fibroblasts and two tumor cell lines in a concentration-dependent fashion (Figure 1A). These three cell lines were compared in terms of integrin content by flow cytometry. Interestingly, the content of α_v_β_3_ is relatively low in these three cell lines compared to the amount of α_2_β_1_. Among the tested cell lines, human fibroblasts are the most sensitive to DisBa-01 and, coincidently, also have the highest content of α_v_β_3_ integrin (Figure 1B). However, a change in receptor density after disintegrin treatment is a possibility that cannot be excluded.

Table 1 summarizes the effects of several disintegrins and DC proteins on tumor-related cell migration in different systems and the range of tested concentrations. The diversity of *in vitro* and *in vivo* assays makes a quantitative comparison difficult. Most tested proteins are effective in a micromolar range for *in vivo* assays while nanomolar concentrations are used in *in vitro* tests. From the data in the literature, it seems that disintegrins are general inhibitors of cell migration and invasion, despite being unspecific ligands, such as the RGD-disintegrins, or integrin-specific binding proteins, such as the KTS-disintegrins (Table 1). The disintegrin-like, Cys-rich proteins may also be included as integrin blockers of cell migration. Since integrin receptors are also quite indiscriminate as they support cell adhesion to several substrates, it seems highly reasonable that the general RGD-disintegrin scaffold of the integrin-binding motif could be employed as a prototype for drug design for new anti-metastatic therapies via blocking both tumor cell adhesion and tumor angiogenesis. In fact, at least two antagonists of platelet α_IIb_β_3_ fibrinogen receptor that are currently being used in anticoagulant therapy (eptifibatide and aggrastat) were based on a snake venom disintegrin structure.

**Table 1:** Structural determinants, preferential integrin and the effects of disintegrins and disintegrin-like proteins on migration-dependent tumor cell activities.

Disintegrin	Structure	Adhesive motif	Preferred integrin	Cognate ligand	Relevant inhibitory activity (conc.*)	Ref.
salmosin 2	monomeric medium	RGD	α_v_β_3_	Vn	angiogenesis ** (5 μg)	[65]
saxatilin	monomeric medium	RGD	α_v_β_3_	Vn	angiogenesis (100 nM)	[54]
jarastatin	monomeric medium	RGD	α_v_β_3_, α_5_β_1_, α_M_β_2_	Vn, Fn, ICAM-1	melanoma lung metastasis ** (1 μM)	[66]
flavoridin	monomeric medium	RGD	α_5_β_1_	Fn	melanoma lung metastasis ** (1 μM)	[66]
kistrin	monomeric medium	RGD	α_v_β_3_	Vn	melanoma lung metastasis **( 1 μM)	[66]
colombistatin	monomeric medium	RGD	nd	Fn	tumor cell migration (IC50 = 1.8 μM)	[67]
trigramin	monomeric medium	RGD	α_v_β_3_	Vn	bone metastasis **(100 μg/ml)	[68]
DisBa-01	monomeric medium	RGD	α_v_β_3_	Vn	melanoma metastasis **(2 mg/Kg)	[33]
eristostatin	monomeric short	RGD	α_IIb_β_3_	Fg	melanoma metastasis (25 μg)	[69]
echistatin	monomeric short	RGD	α_v_β_3_	Vn	osteoclast migration (10 nM)	[70]
triflavin	monomeric short	RGD	α_v_β_3_	Vn	angiogenesis **(0.1–0.4 μM)	[48]
contortrostatin	Homodimeric	RGD	α_5_β_1_, α_v_β_5_	Fn	tumor angiogenesis **(60 μg/day)	[71]
alternagin-C	monomeric D/C	ECD	α_2_β_1_	collagen I	angiogenesis **(1 μM)	[63]
leberagin-C	monomeric D/C	ECD	α_v_β_3_, α_5_β_1_, α_v_β_6_	Vn, Fn	melanoma cell adhesion (100 nM)	[72]
acurhagin-C	monomeric D/C	ECD	α_v_β_3_	Vn	angiogenesis **(0.4 μM)	[61]
VLO5	heterodimeric	VGD, MLD	α_9_β_1_	TN, VCAM	glioblastoma growth (100 μg/ml)	[73]
obtustatin	monomeric short	KTS	α_1_β_1_	collagen IV	angiogenesis **(0.4 μg/μl)	[74]
viperistatin	monomeric short	KTS	α_1_β_1_	collagen IV	melanoma cell transmigration (1–4 μM)	[75]
lebestatin	monomeric short	KTS	α_1_β_1_	collagen IV	angiogenesis **(0.1–0.5 μg/embryo)	[59]
Legend: Vn, vitronectin; Fn, fibronectin; Fg, fibrinogen; TN, tenascin; ICAM-1, intercellular cell adhesion molecule-1; VCAM, vascular cell adhesion molecule. * Effective inhibitory concentration; ** *in vivo* assays.						

It has been suggested that the adhesive properties of the disintegrin-like, Cys-rich proteins could be primarily due to the Cys-rich domain, which was demonstrated to bind vWF [30,76]. Interestingly, half of integrin α subunits possess a von Willebrand factor A domain (also called I domain), which is in close proximity to the ligand-binding site [77]. Therefore, it is possible that the DC proteins could bind to the integrins presenting this domain, thus increasing specificity. However, this possibility remains to be demonstrated.

## 8. ADAMs and Cell Migration

Studies of the snake venom disintegrin effects on cell migration were conducted in parallel with those on mammalian ADAMs. The ADAM (***A*** ***D***isintegrin ***A***nd ***M***etallopeptidase) family of proteins comprises a snake venom-homolog group of multidomain proteins that play important roles in many biological processes, including cell migration, development and fertilization [78,79,80,81,82]. Structurally, the ADAM disintegrin and Cys-rich domains are more related to the DC proteins from SVMPs than the RGD-disintegrins. The disintegrin and Cys-rich domains of ADAM9 were shown to increase keratinocyte migration and MMP-9 activity in a wound healing *in vitro* model, an effect mediated by β1 integrin receptors [83]. More recently, it was reported that knockout animals for ADAM9 showed accelerated wound repair compared to controls, due apparently to the increased migration of keratinocytes [84]. Interestingly, ADAM15, the unique RGD-disintegrin among the ADAMs, suppressed CHO cell motility by inducing integrin α_5_β_1_ expression on the cell surface, thereby enhancing cell adhesion [85]. Additionally, it was demonstrated that the recombinant disintegrin domain of ADAM15 completely inhibited endothelial cell migration and tube formation in a three-dimensional fibrin gel [86]. More recently, the recombinant disintegrin domain of ADAM9 was demonstrated to strongly inhibit MDA-MB-231 breast tumor cell invasion on matrigel *in vitro* [87], thus suggesting a key role for this domain in the process of tumor cell invasion. Moreover, ADAM9 silencing completely inhibited breast tumor cell *in vitro* invasion on matrigel [88]. Recently, it was demonstrated that *in vivo* gene silencing of ADAM9 reduced tumor metastasis [89]. These studies and others in the field with different ADAMs [90,91,92], reinforce the importance of integrins as critical targets for drug design, as well as the potential of disintegrins as lead pharmaceutical compounds.

## 9. Concluding Remarks

Cell adhesion and migration are crucial steps for metastasis development, processes in which the integrins are strongly involved. Snake venom disintegrins have been shown to inhibit metastasis by effectively blocking integrin activities. Apparently, the lack of specificity of these molecules seems to be a positive factor in inhibition of cell adhesion. However, tumor cells have demonstrated their ability to overcome FAK-dependent anchorage and the subsequent anoikis by activating parallel routes such as those mediated by Src and p130CAS [4,93]. It would be interesting to determine if disintegrins can also block these pathways in order to inhibit tumor metastasis completely. A search for new molecules that impair other survival mechanisms of tumor cells may be necessary to achieve improved results in anti-metastatic therapy. Also fascinating is the possible ability of disintegrins to interfere with collective cell migration, which differs from the single cell process mainly by the fact that cells remain coupled by cell-cell junctions while moving. This process depends on simultaneous coordination of cell polarization and was demonstrated to be relevant for cancer invasion and metastasis [94,95,96]. The potential for disintegrins to inhibit collective tumor cell migration remains to be determined.
